# Engineering *Escherichia coli* Biofilms for Curcumin Production

**DOI:** 10.3390/molecules30092031

**Published:** 2025-05-02

**Authors:** Ana Azevedo, Rita Teixeira-Santos, Luciana C. Gomes, Sofia O. D. Duarte, Gabriel A. Monteiro, Filipe J. Mergulhão

**Affiliations:** 1 LEPABE—Laboratory for Process Engineering, Environment, Biotechnology and Energy, Faculty of Engineering, University of Porto, Rua Dr. Roberto Frias, 4200-465 Porto, Portugal; up201407568@edu.fe.up.pt (A.A.); ritadtsantos@fe.up.pt (R.T.-S.); luciana.gomes@fe.up.pt (L.C.G.); 2 ALICE—Associate Laboratory in Chemical Engineering, Faculty of Engineering, University of Porto, Rua Dr. Roberto Frias, 4200-465 Porto, Portugal; 3 iBB—Institute for Bioengineering and Biosciences, Department of Bioengineering, Instituto Superior Técnico, University of Lisbon, 1049-001 Lisboa, Portugal; sofia.duarte@tecnico.ulisboa.pt (S.O.D.D.); gabmonteiro@tecnico.ulisboa.pt (G.A.M.); 4 Associate Laboratory i4HB—Institute for Health and Bioeconomy at Instituto Superior Técnico, Universidade de Lisboa, Av. Rovisco Pais, 1049-001 Lisboa, Portugal

**Keywords:** recombinant protein, biofilm, *Escherichia coli*, curcumin, biosynthetic pathway

## Abstract

Biofilms are emerging platforms for the production of valuable compounds. The present study is the first to assess the capacity of *Escherichia coli* biofilms to produce curcumin through the expression of a biosynthetic pathway involving three genes: 4-coumarate-CoA ligase (*4CL*), diketide-CoA synthase (*DCS*), and curcumin synthase (*CURS*). The effects of chemical induction with isopropyl β-d-1-thiogalactopyranoside (IPTG) and ferulic acid (FA), and the incubation temperature on biofilm formation and curcumin production were evaluated. Biofilms were formed in 12-well microtiter plates over three days and then induced with 1 mM IPTG and FA at 2 or 8 mM. After induction, the samples were incubated for two days at 26 or 30 °C. Total and culturable planktonic and biofilm cells, as well as biofilm thickness and volumetric and specific curcumin production, were assessed on days 3, 4, and 5. The results demonstrated that biofilms produced up to 10-fold higher curcumin levels (0.9–2.2 fg·cell^−1^) than their planktonic counterparts (0.1–0.3 fg·cell^−1^). The highest specific curcumin production (2.2 fg·cell^−1^) was achieved using 8 mM FA. However, no significant differences in curcumin production were observed between the induced samples incubated at the tested temperatures. These results validated the potential of biofilm systems for expressing a complete exogenous biosynthetic pathway using metabolic engineering, particularly for curcumin production.

## 1. Introduction

Curcumin is a yellow natural polyphenol found in the rhizome of *Curcuma longa* and it is part of a group of compounds known as curcuminoids, which include curcumin, demethoxycurcumin, and bisdemethoxycurcumin [1,2]. Curcumin has gained significant attention due to its numerous therapeutic properties, such as antioxidant and anti-inflammatory benefits [2,3]. However, obtaining pure curcumin in a cost-effective and environmentally sustainable manner remains a challenging. To address this, synthetic biology and metabolic engineering approaches have been explored [4,5,6,7,8,9]. Katsuyama et al. [4] first described the production of curcumin using an artificial three-step plasmid biosynthesis pathway within *Escherichia coli* BLR(DE3) involving the 4-coumarate-CoA ligase gene from *Lithospermum erythrorhizon (Le4CL*), the curcuminoid synthase gene from *Oryza sativa* (*OsCUS*), and the acetyl-CoA carboxylase gene from *Corynebacterium glutamicum* (*CgACC*). This approach successfully produced 113 mg·L^−1^ of curcumin from 1 mM ferulic acid (FA). FA is a phenolic compound that has been reported as one of the precursors for the production of curcumin in *E. coli* cells [6]. Subsequent studies were performed to maximize the potential of this biosynthetic pathway [5,6,7,8,9,10], and a two-step plasmid using the *4CL* gene from *Arabidopsis thaliana* (*At4Cl1*), and diketide-CoA synthase and curcumin synthase genes from *Curcuma longa* (*ClDCS* and *ClCURS1*, respectively) in *E. coli* BL21(DE3) was used to produce 563 mg·L^−1^ of curcumin using 3 mM of FA [7]. Recently, Chen et al. [11] performed genome modification of *E. coli* BL21(DE3) including the entire curcumin pathway. This approach used glucose as the primary precursor without the requirement of external precursors. Furthermore, a three-stage pH alternation strategy was employed, leading to a maximum yield of 696 ± 21 mg·L^−1^ of curcumin. Although the integration of the metabolic pathway into the genome can reduce the metabolic burden often associated with plasmid-based systems, the pH alternation strategy played a significant role in increasing curcumin production. Curcumin production in different hosts has been studied by Rainha et al. [12] using *Saccharomyces cerevisiae* and producing 2.7 mg·L^−1^ of curcumin from 30 mg·L^−1^ of FA. Subsequently, Rainha et al. [13] integrated the biosynthetic pathway into the yeast genome obtaining 762.0 ± 59.2 µg·L^−1^ of curcumin from 30 mg·L^−1^ of FA.

Metabolic engineering of *E. coli* connected to recombinant protein (RP) expression enables the production of key enzymes that are part of an exogenous biosynthetic pathway able to synthesize curcumin. These proteins are obtained by isolating a specific gene that encodes the target protein and inserting it into an expression vector system, followed by transformation into the desired host cell [14,15]. Most studies on RPs have focused on their production using planktonic cells [4,6,7]. However, some studies have explored their production using biofilms [16,17,18,19], which consist of structured communities of microorganisms embedded in a self-produced matrix of extracellular polymeric substances [20,21].

In planktonic cells, the expression of RPs imposes a metabolic burden on the host cell [22], as the replication of plasmid DNA and protein synthesis require additional energy and metabolites [14,15,22,23]. This potentially leads to slower cellular growth, reduced biomass yield, and increased plasmid instability, ultimately affecting the yield and activity of the target protein [17,22,24,25]. In turn, cells in biofilms grow slower than their planktonic counterparts [18], allocating fewer resources for replication, which reduces the metabolic burden associated with plasmid maintenance [16]. In addition, the presence of expression vectors in bacterial cells has been shown to increase biofilm formation [17] due to the metabolic stress caused by recombinant gene expression, which may also enhance the production of the target protein compared to their planktonic counterparts [16,17,18].

RP production in bacterial biofilms has advanced with the production of β-galactosidase [25,26,27] and enhanced Green Fluorescent Protein (eGFP) [17,18,19]. Our research group has investigated the expression of eGFP from the pFM23 plasmid in *E. coli* JM109(DE3) strain in both planktonic and biofilm states, exploring the effects of operational parameters on the RP expression and biofilm formation [17,18,19,28]. Indeed, eGFP has been successfully produced in biofilm systems with higher specific production than in planktonic cells. Recently, biofilm-based systems have been employed for the continuous production of human epidermal growth factor (hEGF), demonstrating their potential for high-value biopharmaceutical production. In *E. coli*, biofilm-mediated secretion of hEGF was achieved, highlighting the advantages of biofilm cultivation for protein expression and secretion [29]. Similarly, *S. cerevisiae* biofilms have been explored for continuous hEGF production, further expanding the applicability of biofilm systems to eukaryotic hosts [30]. These advancements underscore the versatility of biofilms for RP production, paving the way for further metabolic engineering innovations.

Although extensive research has been conducted on the production of heterologous compounds through the expression of artificial biosynthetic pathways, such as curcuminoids [6], the potential of biofilms for this purpose remains largely unexplored. In a study by Wigneswaran et al. [31], *Pseudomonas putida* was engineered to produce rhamnolipids by introducing *rhlAB* genes from *Pseudomonas aeruginosa* under synthetic promoters, enabling heterologous biosynthesis in biofilm cells. This biofilm-based system facilitated continuous rhamnolipid production without compromising biofilm integrity, highlighting its potential for sustainable biosurfactant production.

Considering the advantages of using biofilm systems to produce RPs, this study is the first to evaluate the ability of *E. coli* JM109 (DE3) biofilms to produce curcumin through an artificial biosynthetic pathway over three days using FA as a precursor. Additionally, as our previous studies denoted that operational conditions affect RP expression [28], the effects of FA concentration and temperature on biofilm development and curcumin production were investigated. The results indicated that biofilms have great potential for curcumin production compared to their planktonic counterparts, and that higher concentrations of FA lead to increased curcumin production, while temperature did not significantly impact production.

## 2. Results and Discussion

Given that our previous studies showed that operational parameters influence biofilm formation and RP production [19,28], the effects of precursor (FA) concentration and temperature on curcumin production yields by both planktonic and biofilm cells were assessed.

### 2.1. The Effect of Ferulic Acid Concentration on Biofilm Formation and Curcumin Production

Since FA is used by *E. coli* as a precursor in the curcumin biosynthesis pathway, it is important to assess the impact of its concentration on biofilm formation and curcumin yield. Katsuyama et al. [1] achieved a curcumin production of 113 mg·L^−1^ using 1 mM FA. Rodrigues et al. [7] evaluated FA concentrations ranging from 1 mM to 4 mM, and reported that the highest curcumin production in planktonic cells (563 mg·L^−1^) was obtained with a final concentration of 3 mM FA. In addition, a study evaluating the antimicrobial activity of FA demonstrated that a 5 mM concentration reduced the growth of 24 h biofilms, although it did not completely remove the biofilm [32].

In our study, biofilms of three days were exposed to 2 mM and 8 mM FA and incubated at 30 °C until day 5. The number of total and culturable planktonic and biofilm cells, as well as the biofilm thickness and curcumin production, were evaluated.

**Figure 1:**
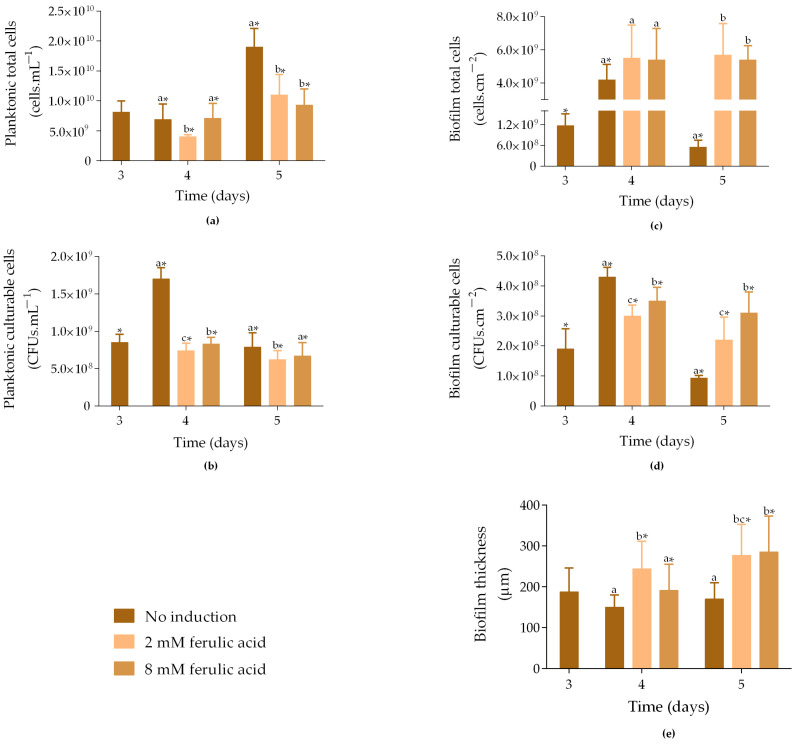
Total and culturable planktonic (**a**,**b**) and biofilm cells (**c**,**d**), and biofilm thickness (**e**) from samples exposed to different concentrations of ferulic acid (FA). Samples were incubated at 30 °C without induction (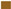), and with 1 mM IPTG and 2 mM FA (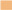) or 8 mM FA (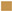) added on day 3. The samples were analyzed on days 3, 4, and 5. The means ± SD for three independent experiments with three technical replicates each are presented. Statistically significant differences within each day (denoted by letters) and between days under the same condition (denoted by *) were considered for *p*-values < 0.05.

On day 3, the non-induced samples exhibited 8.2 × 10^9^ cells·mL^−1^ (Figure 1a). On day 4, both non-induced samples and those induced with IPTG and 8 mM FA exhibited a similar average number of planktonic cells (7.0 × 10^9^ cells·mL^−1^). In contrast, samples induced with IPTG and 2 mM FA displayed 42% fewer cells compared to non-induced samples on the same day (*p* < 0.05). On day 5, non-induced samples showed a 63% increase in the number of total cells compared to day 4 (*p* < 0.05), while induced samples exhibited a slight increase in cell number (24% for 8 mM FA and 36% for 2 mM FA; *p* < 0.05), reaching approximately 1.1 × 10^10^ cells·mL^−1^. These data suggest that simultaneous exposure to IPTG and FA negatively affected *E. coli* planktonic growth, regardless of the FA concentration used.

Regarding planktonic culturable cells, on day 3, non-induced samples had, on average, 8.5 × 10^8^ CFUs·mL^−1^ (Figure 1b), which increased by 50% on day 4 (*p* < 0.05). In contrast, induced samples showed slightly decreased culturability throughout the assay (*p* < 0.05), reaching 6.7 × 10^8^ CFUs·mL^−1^ for samples exposed to 8 mM FA and 6.2 × 10^8^ CFUs·mL^−1^ for those exposed to 2 mM FA. Therefore, as observed for total cells (Figure 1a), the addition of FA negatively affected the culturability of planktonic cells, particularly at a concentration of 2 mM.

Concerning biofilm cells, on day 3, non-induced samples showed, on average, 1.2 × 10^9^ cells·cm^−2^ (Figure 1c). The number of total cells increased by 3.6-fold on day 4 (*p* < 0.05) and was similar to that of the induced samples. However, on day 5, non-induced samples showed an 87% decrease in the number of cells compared to day 4 (*p* < 0.05), while induced samples were able to maintain the number of cells (5.4 × 10^9^ and 5.7 × 10^9^ cells·cm^−2^) under exposure to either 8 mM or 2 mM FA. This suggests that biofilm growth was not negatively affected by FA addition. Furthermore, biofilm culturable cells increased by 66% from day 3 to day 4, and then decreased by 78% on day 5 (Figure 1d; *p* < 0.05). On day 4, samples induced with IPTG and 8 mM FA exhibited 3.5 × 10^8^ CFUs·cm^−2^, and samples induced with IPTG and 2 mM presented 3.0 × 10^8^ CFUs·cm^−2^. On day 5, although the number of culturable cells was higher when compared to non-induced samples, it decreased by 11% and 27% in samples induced with 8 mM and 2 mM FA, respectively, compared to day 4 (*p* < 0.05).

Biofilm thickness followed the same trend observed for the biofilm total cell count, with induced biofilms exhibiting higher thickness compared to non-induced biofilms on both days 4 and 5 (Figure 1e). The thickness of non-induced biofilms slightly decreased from day 3 to day 4, without statistically significant differences, indicating that the biofilms were stable during this period.

In general, these data demonstrated that the growth and culturability of planktonic cells were negatively affected by induction exposure, while biofilms were not. It is important to note that the negative impact of induction on planktonic cells may be related to the presence of IPTG, which has been shown to impose metabolic stress on cells [19]. Additionally, although previous studies have shown that 5 mM FA inhibits biofilm growth [32], our results indicate that both tested concentrations enhanced biofilm development compared to non-induced samples. The same behaviour was observed when analyzing the effects of IPTG alone (1 mM) and FA alone at both concentrations (2 and 8 mM) on biofilm formation (Figure S1 in Supplementary Material). In general, any of these conditions led to an increase in sessile cell numbers and biofilm thickness compared to non-induced biofilms (control), supporting the idea that neither IPTG nor FA exhibits toxic effects.

The increased biofilm cell growth upon FA supplementation may be attributed to several factors, including extracellular polymeric substance (EPS) production, oxidative stress responses, and quorum sensing (QS) modulation. EPS can act as a diffusion barrier, modulating the penetration of compounds such as FA, and allowing sessile cells to develop tolerance and adapt to potentially stressful conditions [33,34]. Additionally, FA-induced oxidative stress may trigger adaptive stress responses and result in enhanced biofilm formation [35]. FA may also influence QS pathways, promoting bacterial adhesion and matrix production [36].

**Figure 2:**
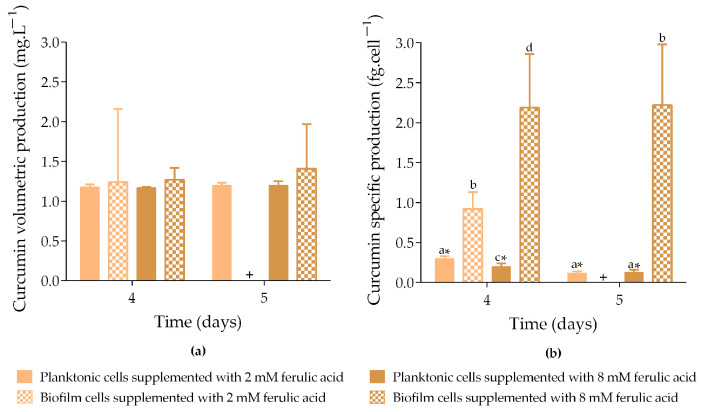
Curcumin volumetric production (**a**) and specific curcumin production (**b**) by planktonic and biofilm cells exposed to different concentrations of ferulic acid (FA). Samples were incubated at 30 °C with 1 mM IPTG and 2 mM FA (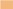 planktonic cells and 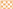 biofilm cells) or 8 mM FA (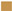 planktonic cells and 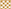 biofilm cells) added on day 3. Curcumin production was analyzed on days 4 and 5. + means that no curcumin production was quantified. The means ± SD for three independent experiments with three technical replicates each are presented. Statistically significant differences within each day (denoted by letters) and between days under the same condition (denoted by *) were considered for *p*-values < 0.05.

The volumetric curcumin production obtained from both planktonic and biofilm cells on days 4 and 5 ranged from 1.2 to 1.4 mg·L^−1^ (Figure 2a). No statistically significant differences were observed between the cell states (planktonic and biofilm) or FA concentrations (2 and 8 mM). However, curcumin was not detected in biofilm cells induced with IPTG and 2 mM FA by day 5.

Concerning specific curcumin production (Figure 2b), biofilms exposed to 8 mM FA produced up to 17-fold more curcumin than planktonic cells exposed to the same conditions (*p* < 0.05; 2.2 fg·cell^−1^ vs. 0.2 and 0.1 fg·cell^−1^, on days 4 and 5, respectively). On day 4, biofilms exposed to 2 mM FA produced 3-fold more curcumin than planktonic cells exposed to the same conditions (*p* < 0.05; 0.9 fg·cell^−1^ vs. 0.3 fg·cell^−1^). The increased curcumin production observed in biofilms exposed to the highest FA concentration tested (8 mM) may be a consequence of a higher consumption of FA through the artificial biosynthetic pathway expressed in *E. coli* cells. This hypothesis was supported by the experimental quantification of the residual FA concentration at the end of the experiment (Table S1 in Supplementary Material). These new data revealed greater precursor conversion when the system was supplemented with 8 mM (on average 95%) than 2 mM (on average 43%).

Moreover, specific production data demonstrated that curcumin was more efficiently produced by biofilm cells compared to planktonic cells. This may be due to the metabolic burden of the At4Cl_DCS_CURS plasmid in planktonic cells, which appears to negatively impact cellular growth and viability under induction conditions (Figure 1a,b). In this growth state, plasmid DNA replication and protein synthesis require additional energy and metabolites, potentially leading to plasmid instability and metabolic alterations that reduce the overall protein yield and activity [4,21,22,23]. In contrast, cells in biofilms grow more slowly than their planktonic counterparts [18], allocating fewer resources for replication and reducing the metabolic burden associated with plasmid maintenance. This may result in greater operational stability and higher production rates [24,25].

Although this study represents a breakthrough in expanding and validating the biofilm system for curcumin production, the volumetric productions obtained here were lower than the 563 mg·L^−1^ reported by Rodrigues et al. [7] using 3 mM FA. This difference in curcumin production may be explained by the genetic approach, host cells, and the operational conditions used. Rodrigues et al. [7] used a two-plasmid approach (pACYCduet_4CL and pRSFduet_CURS1_DCS), which were transformed into the mutant strain *E. coli* BL21(DE3)ΔlacZ. Bacterial cells were initially grown in Lysogeny Broth (LB) medium at 37 °C until they reached an OD_600_ of 0.9. At this time point, cells were induced with 0.1 mM IPTG for plasmid expression and incubated for 5 h at 26 °C. Afterwards, cells were resuspended in M9 medium, induced with 0.1 mM IPTG, and supplemented with 3 mM FA. FA was added in two phases: 2 mM at the time of induction and 1 mM after 15 h. Curcumin production was conducted at 26 °C for 63 h. Comparatively, our study was conducted using a one-plasmid approach, which could be an effective strategy to reduce the metabolic burden. In addition, the IPTG concentration and culture medium were distinct, which may also influence the curcumin production levels. As we previously reported, the composition of the culture medium significantly impacts recombinant protein expression [28]. Lastly, the higher curcumin production achieved by Rodrigues et al. [7] was obtained using a two-step addition of the precursor FA. Taking these differences into account, further optimization can focus on the host strain, plasmid design, and cultivation conditions (such as the precursor addition regimen) to enhance curcumin production in biofilm systems.

### 2.2. The Effect of Incubation Temperature on Curcumin Production

It has been shown that *E. coli* typically has an optimal growth temperature of 37 °C [37], and most *E. coli* biofilm formation studies have been performed within a temperature range of 30–37 °C [16,19,25,26,27]. However, curcumin production using planktonic *E. coli* cells has mostly been performed at 26 °C [4,6,7]. Furthermore, Kang et al. [38] reported that a temperature of 26 °C was better for curcumin production than 37 °C.

To evaluate the influence of the incubation temperature on curcumin production, *E. coli* biofilms were induced on day 3 of formation with 1 mM IPTG and 8 mM FA (the FA concentration which led to higher levels of curcumin; Figure 2b) and incubated at 26 °C or 30 °C for more two days. The number of total and culturable planktonic and biofilm cells, as well as the biofilm thickness and curcumin production, were evaluated.

**Figure 3:**
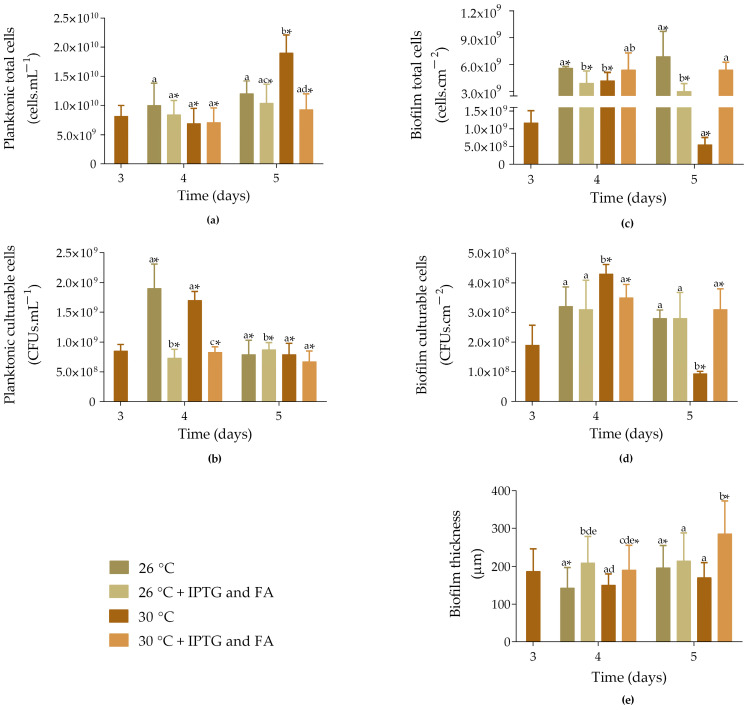
Total and culturable planktonic (**a**,**b**) and biofilm cells (**c**,**d**), and biofilm thickness (**e**) of samples incubated at different temperatures. Non-induced (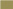 and 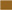) and induced samples (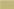 and 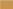) with 1 mM IPTG and 8 mM FA on day 3 and incubated at 26 and 30 °C. The samples were analyzed on days 3, 4, and 5. The means ± SD for three independent experiments with three technical replicates each are presented. Statistically significant differences within each day (denoted by letters) and between days under the same condition (denoted by *) were considered for *p*-values < 0.05.

On day 3, the non-induced samples exhibited an average of 8.2 × 10^9^ cells·mL^−1^ (Figure 3a). On day 4, the number of total cells slightly decreased, and was similar for non-induced and induced samples, regardless of whether they were incubated at 26 °C or 30 °C. In turn, on day 5, the number of cells increased, with non-induced samples incubated at 30 °C showing the highest cell count (1.9 × 10^10^ cells·mL^−1^). Additionally, induced samples incubated at 26 °C exhibited 1.2-fold more cells compared to those incubated at 30 °C (*p* < 0.05).

Concerning planktonic culturable cells, on day 4, the number of cells was higher for non-induced samples incubated at 26 °C and 30 °C (1.9 × 10^9^ and 1.7 × 10^9^ CFUs·mL^−1^; *p* < 0.05; Figure 3b). In turn, induced samples exhibited 7.3 × 10^8^ CFUs·mL^−1^ when incubated at 26 °C and 8.3 × 10^8^ CFUs·mL^−1^ when incubated at 30 °C (*p* < 0.05). On day 5, the number of culturable cells was very similar across the different growth conditions, with the highest values observed for induced samples incubated at 26 °C (8.8 × 10^8^ CFUs·mL^−1^; *p* < 0.05).

Biofilm total cells showed a significant increase from day 3 to 4 for all four samples tested (Figure 3c; *p* < 0.05), with non-induced samples incubated at 26 °C and induced samples incubated at 30 °C showing a higher number of cells (5.6 × 10^9^ cells·cm^−2^ and 5.4 × 10^9^ cells·cm^−2^, respectively). On day 5, this behaviour was maintained.

The number of biofilm culturable cells significantly increased from day 3 to 4 for both non-induced and induced samples incubated at 30 °C (Figure 3d; *p* < 0.05). However, on day 5, the cell counts significantly decreased in these samples by 78% and 11%, respectively (*p* < 0.05). Furthermore, no significant differences were observed between the induced samples incubated at 26 °C and 30 °C (2.8 × 10^8^ cells·cm^−2^ and 3.1 × 10^8^ cells·cm^−2^, respectively).

Concerning biofilm thickness, induced samples incubated at either 26 °C or 30 °C were approximately 30% thicker than the non-induced samples on day 4 (Figure 3e). In turn, on day 5, the induced samples incubated at 30 °C showed an increase in thickness, recording the highest value (285 ± 87 µm; *p* < 0.05), which corroborates the results obtained from the total cell count. In general, induced biofilms incubated at 30 °C exhibited a higher number of cells and greater thickness compared to those incubated at 26 °C.

**Figure 4:**
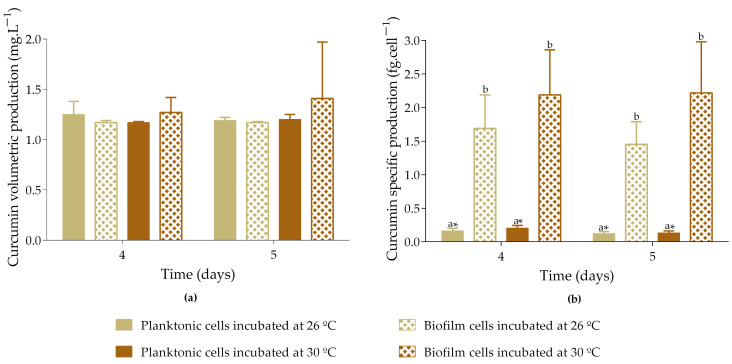
Volumetric curcumin production (**a**) and specific curcumin production (**b**) by planktonic and biofilm cells incubated at different temperatures. Samples induced with 1 mM IPTG and 8 mM FA on day 3 were incubated at 26 or 30 °C. Curcumin production was analyzed on days 4 and 5. The means ± SD for three independent experiments with three technical replicates each are presented. Statistically significant differences within each day (denoted by letters) and between days under the same condition (denoted by *) were considered for *p*-values < 0.05.

The volumetric production of curcumin ranges from 1.2 to 1.4 mg·L^−1^, with no significant differences observed between the planktonic and biofilm cells incubated at either 26 °C or 30 °C (Figure 4a). However, specific production analysis revealed that biofilm cells produced significantly more curcumin than planktonic cells, with production levels up to 12-fold higher for cells incubated at 26 °C and 17-fold higher for those incubated at 30 °C (Figure 4b). Although there were no significant differences in the specific curcumin production by biofilm cells incubated at 26 °C and 30 °C, there was a tendency for cells incubated at 30 °C to produce higher levels of curcumin (2.2 fg·cell^−1^ vs. 1.7 fg·cell^−1^), which may be associated with the optimal temperature for *E. coli* biofilm development.

Biofilm systems face challenges, such as limited oxygen and substrate diffusion, which can increase population heterogeneity, the complexity of maintaining a pure culture in consecutive operations, and difficulties associated with scaling up and purifying intracellular products [39,40,41]. However, despite the need for optimization of operational conditions, genetic approach and host cells, biofilm systems present several advantages compared to traditional free-cell fermentation systems, as they are able to retain more biomass per unit volume, can resist stress conditions, have a reduced risk of washout, which eliminates the need for repeated inoculations, present higher production rates and yields, and exhibit long-term activity [39,41,42,43], making them a promising platform to produce value-added compounds.

## 3. Materials and Methods

### 3.1. Plasmid Construction

For plasmid construction, the pET28a vector was selected as the backbone due to its robust transcription control via the T7 promoter and its compatibility with high-yield protein expression systems [44]. It contains a kanamycin resistance (*KanR*) gene, a pMB1 origin of replication (medium-copy number replicon), and utilizes the T7 promoter for gene transcription, which can be induced by isopropyl β-D-1-thiogalactopyranoside (IPTG) [45,46]. The genes 4-coumarate-CoA ligase (*At4Cl*) from *Arabidopsis thaliana*, and diketide-CoA synthase (*DCS*) and curcumin synthase (*CURS*) from *Curcuma longa* were selected to express the curcumin biosynthetic pathway in *E. coli*. The At4Cl_DCS_CURS plasmid was then designed (Figure S2 in Supplementary Material) and assembled by NZYTech (Lisboa, Portugal).

**Figure 5:**
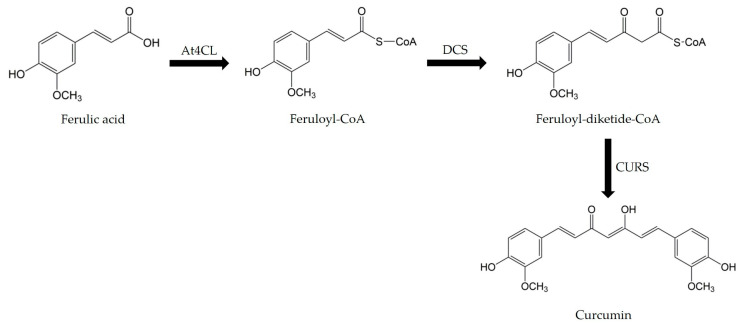
Curcumin biosynthetic pathway.

In this pathway, FA is used as a precursor and is first converted to feruloyl-CoA by 4-coumarate-CoA ligase (4CL). Feruloyl-CoA is then converted to feruloyl-diketide-CoA by feruloyl-diketide-CoA synthase (DCS), and finally, curcumin is formed from feruloyl-diketide-CoA by the curcumin synthase (CURS) (Figure 5).

### 3.2. Bacterial Strain and Growth Conditions

The *E. coli* strain JM109(DE3) (Promega, Madison, WI, USA) was transformed by heat shock with the At4Cl_DCS_CURS plasmid for the production of curcumin [47]. Transformant bacteria were stored at –80 °C in Lysogeny Broth medium (LB; Sigma-Aldrich, St. Louis, MO, USA) with 30% (*v*/*v*) glycerol. Before the experiments, bacteria were spread on Plate Count Agar (PCA, Merck, Darmstadt, Germany) supplemented with 20 µg·mL^−1^ kanamycin (Eurobio, Les Ulis, France) to select transformant cells and incubated overnight (16–18 h) at 30 °C.

Cell suspensions were prepared by inoculating 1–2 bacterial colonies into 100 mL of Terrific Broth (TB) supplemented with 20 µg·mL^−1^ kanamycin. TB medium contains per litre: 12.0 g bacto tryptone (VWR BDH chemicals, Radnor, PA, USA), 24.0 g yeast extract (Sigma-Aldrich, St. Louis, MO, USA), 4.0 mL glycerol (VWR BDH chemicals, Radnor, PA, USA), 2.3 g KH_2_PO_4_ (VWR BDH chemicals, Radnor, PA, USA), and 12.5 g K_2_HPO_4_ (Sigma-Aldrich, St. Louis, MO, USA).

Bacterial cultures were grown overnight at 30 °C and 120 rpm (Agitorb 200ICP, Norconcessus, Ermesinde, Portugal), and then adjusted to an optical density at 610 nm (OD_610_) of 0.15, corresponding to 1 × 10^8^ cells·mL^−1^.

### 3.3. Biofilm Formation and Curcumin Production

**Figure 6:**
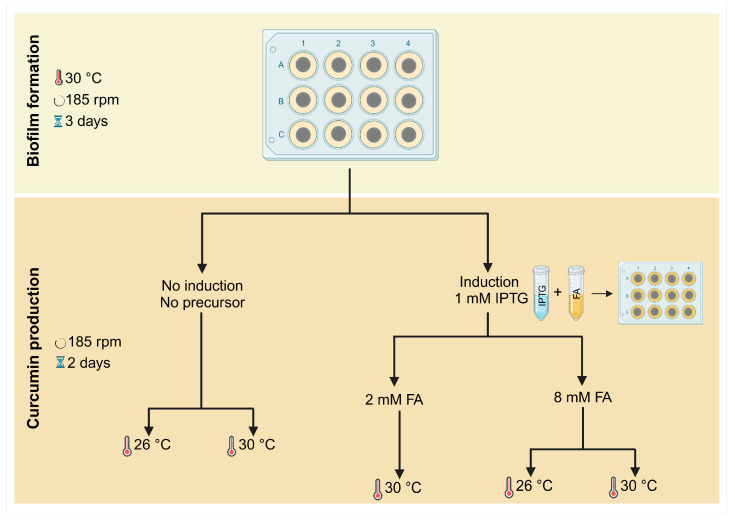
Representative workflow of biofilm formation and curcumin production over five days.

Biofilms of *E. coli* transformed with the At4Cl_DCS_CURS plasmid were formed on 12-well microplates (MTPs, VWR International, Carnaxide, Portugal) over five days, following the scheme shown in Figure 6.

Circular coupons of polyvinyl chloride (PVC) with a 1 cm diameter were used for biofilm formation assays. PVC is a synthetic polymer commonly used in industrial settings, and recent studies have shown that it is a good material for biofilm formation [28]. PVC coupons were washed by immersion in ethanol 96% (*v*/*v*) for 1 h with gentle shaking, then rinsed with ultrapure water for 1 h and autoclaved for 20 min at 70 °C, as previously described [48]. They were then fixed to the microplate wells using double-sided adhesive tape and subjected to ultraviolet sterilization for 1 h. Subsequently, the coupons were inoculated with 3 mL of bacterial suspension containing 1 × 10^8^ cells·mL^−1^. The microplates were incubated at 30 °C and 185 rpm for three days to allow biofilm formation (Figure 6, top), as previously described [28].

For curcumin production, samples were induced on day 3 with 1 mM IPTG (BIORON GmbH, Ludwigshafen, Germany), supplemented with either 2 mM or 8 mM FA (Sigma-Aldrich, St. Louis, MO, USA), and incubated at 30 °C for more two days. Also, the impact of IPTG alone (1 mM) and FA alone (2 mM or 8 mM) on biofilm formation was assessed (Figure S1 in Supplementary Material).

Additionally, to evaluate the effect of temperature on curcumin production, samples induced with 1 mM IPTG and supplemented with 8 mM FA were also incubated at 26 °C (Figure 6, bottom). Samples without induction and FA supplementation, incubated at either 26 °C or 30 °C, served as controls.

The culture medium was replaced on days 1 and 3, and sampling was performed on days 3, 4, and 5. All experiments were conducted in triplicate, with three technical replicates for each condition.

### 3.4. Sample Analysis

On each sampling day, 3 mL of culture medium containing the planktonic cells was collected from each well and transferred into Falcon tubes. The PVC coupons were then removed from the MTP wells, placed in sterile sodium chloride solutions (NaCl, 8.5 g·L^−1^), and vortexed at maximum speed for 3 min to obtain biofilm cell suspensions. The number of total and culturable planktonic and biofilm cells, as well as curcumin production by both types of cells, were quantified. Additionally, the biofilm thickness was assessed using Optical Coherence Tomography (OCT).

#### 3.4.1. Total Cell Count

Total cells were quantified using a CytoFLEX flow cytometer model V0 B3-R1 (Beckman Coulter, Brea, CA, USA) with CytExpert software (version 2.4.0.28, Beckman Coulter, Brea, CA, USA). Bacteria were gated based on their side scatter (SSC) and forward scatter (FSC) signals.

Sample acquisition was conducted at a flow rate of 10 µL·min^−1^, and 10 µL of bacterial suspension was acquired. The results are presented as cells·mL^−1^ for the planktonic state and cells·cm^−2^ for the biofilm state.

#### 3.4.2. Culturable Cell Count

The number of culturable cells was determined by colony-forming unit (CFU) count. Serial dilutions of planktonic and biofilm suspensions were performed and spread on PCA supplemented with 20 µg·mL^−1^ kanamycin and incubated overnight at 30 °C for colony enumeration. The results are presented as CFUs·mL^−1^ for the planktonic state and CFUs·cm^−2^ for the biofilm state.

#### 3.4.3. Curcumin Extraction

Curcumin extraction was performed as described by Rodrigues et al. [7]. Briefly, the pH of each sample was adjusted to 3.0 using 1.5 M HCl. An equal volume of ethyl acetate (Supelco, Bellefonte, PA, USA) was added to the samples and homogenized for 1 min using a vortex. Subsequently, the aqueous and organic phases were separated by centrifugation at 17,000× *g* for 5 min. The top organic phase was collected in a new tube, and the extracts were concentrated by solvent evaporation in a fume hood. The extracts were resuspended in 200 µL of acetonitrile (VWR, Radnor, PA, USA) and centrifuged for 6000× *g* for 3 min to separate any residual debris. Approximately 150 µL of the extract was placed in the high-performance liquid chromatography (HPLC) microinserts (VWR, Radnor, PA, USA).

#### 3.4.4. Curcumin and Ferulic Acid Quantification

Curcumin and residual FA quantification was performed using HPLC analysis with a Nexera-I LC-2040C 3D system (Shimadzu, Duisburg, Germany) and a Macherey-Nagel C18 column (5 μm, 250 mm × 4 mm, 250 Å).

Calibration curves for curcumin and FA were constructed by performing serial dilutions of a FA stock solution (10 mg·mL^−1^; Sigma-Aldrich, St. Louis, MO, USA) and a curcumin stock solution (10 mg·mL^−1^; Tokyo Chemical Industry Co., Belgium, Europe) using acetonitrile. The resulting FA (250, 100, 50, 25, 12.5, 6.25, 3.125, 1.56, 0.78, 0.39, 0.19 µg·mL^−1^) and curcumin solutions (100, 50, 25, 12.5, 6.25, 3.125, 1.56, 0.78, 0.39, 0.19 µg·mL^−1^) were then analyzed using HPLC.

The chromatography protocol analysis was set at 15 min, at a flow rate of 1 mL·min^−1^, with a mobile phase of 50% H_2_O and 50% acetonitrile with 0.1% formic acid. The pH was adjusted to a range between 4.0 and 5.0, and the detection was performed at 310 nm and 425 nm with a retention time of 2.4–2.7 min and 10.0–10.7 min for FA and curcumin, respectively.

#### 3.4.5. Biofilm Thickness

Biofilm thickness was determined through OCT (Thorlabs Ganymede Spectral Domain Optical Coherence Tomography system, Thorlabs GmbH, Dachau, Germany). Before biofilm analysis, the culture medium was carefully removed from the microplate wells to avoid disrupting the biofilms. Subsequently, the coupons were gently washed, and the wells were filled with a sterile solution of 8.5 g·L^−1^ NaCl. The refractive index was set to 1.40 (similar to that of water, which is 1.33), and a minimum of five different fields of view (2D images) were captured for each sample. The processing and analysis of OCT images were performed using a routine developed in the Image Processing Toolbox from MATLAB 8.0 and Statistics Toolbox 8.1 (The MathWorks, Inc., Natick, MA, USA) [49].

### 3.5. Statistical Analysis

Statistical analysis was performed using the IBM SPSS Statistics version 28 for Windows (IBM SPSS, Inc., Chicago, IL, USA). Descriptive statistics were used to calculate the mean and standard deviation (SD) for the number of planktonic and biofilm total and culturable cells, curcumin volumetric and specific productions, and biofilm thickness.

Since the variables were not normally distributed, a nonparametric analysis using the Kruskal–Wallis test was performed to assess differences among groups. Differences between groups were determined using the Mann–Whitney test.

Statistically significant differences (*p* < 0.05) are indicated by different lowercase letters within days, and by * between days for the same condition. All data are presented as mean ± standard deviation (SD) from three independent experiments with three technical replicates each.

## 4. Conclusions

This study demonstrated for the first time that *E. coli* biofilms can produce curcumin through the expression of a complete biosynthetic pathway containing genes from different organisms. It was also demonstrated that under the experimental conditions used, specific production yields were higher in biofilms than in planktonic cells. This raises the possibility of using *E. coli* biofilms to produce different value-added compounds, including production strategies with multispecies biofilms, where cells containing different genetic elements may be used collaboratively to produce complex molecules. The spontaneous association of different bacteria in environmental biofilms, where some species benefit from the metabolites produced by others, can be considered a template for the heterologous production of complex molecules in biofilm systems.

The analysis of planktonic cells revealed that induction with IPTG and ferulic acid negatively affected *E. coli* growth. In contrast, biofilm development was not negatively affected by the addition of IPTG and FA. Furthermore, curcumin production increased when a higher concentration of FA was used. Concerning incubation temperature, data indicated that induced biofilms incubated at 30 °C exhibited a higher number of cells and greater thickness compared to those incubated at 26 °C. However, no significant differences in curcumin production were observed between induced samples incubated at 26 °C and 30 °C.

Although these findings are crucial for achieving cost-effective curcumin production, lower protein levels were obtained compared to previous studies using different approaches and experimental conditions. This suggests that certain operational parameters, including the precursor addition regimen and concentration, should be optimized in future experiments aimed at increasing curcumin production using biofilm systems. Future studies should consider scaling up this approach by using a continuous system while monitoring ferulic acid consumption and curcumin production.

## Data Availability

The original contributions presented in the study are included in the article and Supplementary Material. Further inquiries can be directed to the corresponding author.

## Supplementary Materials

The following supporting information can be downloaded at https://www.mdpi.com/article/10.3390/molecules30092031/s1, Figure S1. Total and culturable biofilm cells, and biofilm thickness from samples exposed to IPTG only (1 mM) and FA only (2 mM and 8 mM). Samples were incubated at 30 °C without induction (), and with 1 mM IPTG (), 2 mM FA (), and 8 mM FA () added on day 3. The samples were analyzed on days 3, 4, and 5. The means ± SD for three independent experiments with three technical replicates each are presented. Statistically significant differences within each day (denoted by letters) and between days under the same condition (denoted by *) were considered for *p*-values < 0.05. Figure S2. Plasmid At4Cl_DCS_CURS map. This harbours (i) a repressor for the lac promoter (lacI), (ii) a lactose operator (lac operator), (iii) a transcriptional promoter from the T7 phage (T7 promoter), (iv) a T7 transcriptional terminator (T7 terminator), (v) a kanamycin resistance gene (*KanR*), and (vi) the three genes selected for the curcumin biosynthetic pathway: 4-coumarate-CoA ligase (*At4Cl*) from *Arabidopsis thaliana*, and diketide-CoA synthase (*DCS*) and curcumin synthase (*CURS*) from *Curcuma longa*. Table S1. Residual FA concentration (mM) upon induction with 1 mM IPTG and 8 mM FA or 2 mM FA on day 3 and incubation at 26 °C or 30 °C until day 5. Data are presented as mean ± SD.

## References

[1] Amalraj A., Pius A., Gopi S., Gopi S. (2017). Biological activities of curcuminoids, other biomolecules from turmeric and their derivatives—A review. J. Tradit. Complement. Med..

[2] Hewlings S.J., Kalman D.S. (2017). Curcumin: A Review of Its Effects on Human Health. Foods.

[3] Hsu K.Y., Ho C.T., Pan M.H. (2023). The therapeutic potential of curcumin and its related substances in turmeric: From raw material selection to application strategies. J. Food Drug Anal..

[4] Katsuyama Y., Matsuzawa M., Funa N., Horinouchi S. (2008). Production of curcuminoids by *Escherichia coli* carrying an artificial biosynthesis pathway. Microbiology.

[5] Wu J., Chen W., Zhang Y., Zhang X., Jin J.M., Tang S.Y. (2020). Metabolic Engineering for Improved Curcumin Biosynthesis in *Escherichia coli*. J. Agric. Food Chem..

[6] Rainha J., Rodrigues L.R., Rodrigues J.L., Jafari S.M., Harzevili F.D. (2022). Microbial Production of Curcumin. Microbial Production of Food Bioactive Compounds.

[7] Rodrigues J.L., Gomes D., Rodrigues L.R. (2020). A Combinatorial Approach to Optimize the Production of Curcuminoids From Tyrosine in *Escherichia coli*. Front. Bioeng. Biotechnol..

[8] Rodrigues J.L., Araujo R.G., Prather K.L., Kluskens L.D., Rodrigues L.R. (2015). Production of curcuminoids from tyrosine by a metabolically engineered *Escherichia coli* using caffeic acid as an intermediate. Biotechnol. J..

[9] Rodrigues J.L., Prather K.L., Kluskens L.D., Rodrigues L.R. (2015). Heterologous production of curcuminoids. Microbiol. Mol. Biol. Rev..

[10] Couto M.R., Rodrigues J.L., Rodrigues L.R. (2017). Optimization of fermentation conditions for the production of curcumin by engineered *Escherichia coli*. J. R. Soc. Interface.

[11] Chen J., Wang W., Wang L., Wang H., Hu M., Zhou J., Du G., Zeng W. (2024). Efficient De Novo Biosynthesis of Curcumin in *Escherichia coli* by Optimizing Pathway Modules and Increasing the Malonyl-CoA Supply. J. Agric. Food Chem..

[12] Rainha J., Rodrigues J.L., Faria C., Rodrigues L.R. (2022). Curcumin biosynthesis from ferulic acid by engineered *Saccharomyces cerevisiae*. Biotechnol. J..

[13] Rainha J., Rodrigues J.L., Rodrigues L.R. (2024). De Novo Biosynthesis of Curcumin in *Saccharomyces cerevisiae*. ACS Synth. Biol..

[14] Overton T.W. (2014). Recombinant protein production in bacterial hosts. Drug Discov. Today.

[15] Rosano G.L., Ceccarelli E.A. (2014). Recombinant protein expression in *Escherichia coli*: Advances and challenges. Front. Microbiol..

[16] O’Connell H.A., Niu C., Gilbert E.S. (2007). Enhanced high copy number plasmid maintenance and heterologous protein production in an *Escherichia coli* biofilm. Biotechnol. Bioeng..

[17] Gomes L., Mergulhão F. (2017). Heterologous protein production in *Escherichia coli* biofilms: A non-conventional form of high cell density cultivation. Process Biochem..

[18] Soares A., Gomes L.C., Mergulhão F.J. (2018). Comparing the Recombinant Protein Production Potential of Planktonic and Biofilm Cells. Microorganisms.

[19] Gomes L., Monteiro G., Mergulhao F. (2020). The Impact of IPTG Induction on Plasmid Stability and Heterologous Protein Expression by *Escherichia coli* Biofilms. Int. J. Mol. Sci..

[20] Flemming H.C., Wingender J., Szewzyk U., Steinberg P., Rice S.A., Kjelleberg S. (2016). Biofilms: An emergent form of bacterial life. Nat. Rev. Microbiol..

[21] Sauer K., Stoodley P., Goeres D.M., Hall-Stoodley L., Burmolle M., Stewart P.S., Bjarnsholt T. (2022). The biofilm life cycle: Expanding the conceptual model of biofilm formation. Nat. Rev. Microbiol..

[22] Hoffmann F., Rinas U. (2004). Stress induced by recombinant protein production in *Escherichia coli*. Adv. Biochem. Eng. Biotechnol..

[23] Sorensen H.P., Mortensen K.K. (2005). Advanced genetic strategies for recombinant protein expression in *Escherichia coli*. J. Biotechnol..

[24] Haddadin F.T., Harcum S.W. (2005). Transcriptome profiles for high-cell-density recombinant and wild-type *Escherichia coli*. Biotechnol. Bioeng..

[25] Huang C.T., Peretti S.W., Bryers J.D. (1994). Effects of medium carbon-to-nitrogen ratio on biofilm formation and plasmid stability. Biotechnol. Bioeng..

[26] Huang C.T., Peretti S.W., Bryers J.D. (1994). Effects of inducer levels on a recombinant bacterial biofilm formation and gene expression. Biotechnol. Lett..

[27] Huang C.T., Peretti S.W., Bryers J.D. (1993). Plasmid retention and gene expression in suspended and biofilm cultures of recombinant *Escherichia coli* DH5alpha(pMJR1750). Biotechnol. Bioeng..

[28] Azevedo A., Teixeira-Santos R., Carvalho F.M., Gomes L.C., Monteiro G.A., Mergulhão F.J. (2024). Influence of Surface Material and Nutrient Conditions on Green Fluorescent Protein Production in *Escherichia coli* Biofilms. Appl. Sci..

[29] Zhang C., Liao J., Li Y., Liu S., Li M., Zhang D., Wang Z., Liu D., Ying H. (2024). Continuous Secretion of Human Epidermal Growth Factor Based on *Escherichia coli* Biofilm. Fermentation.

[30] Zhi K., An Z., Zhang M., Liu K., Cai Y., Wang Z., Zhang D., Liu J., Wang Z., Zhu C. (2024). Biofilm-Based Immobilization Fermentation for Continuous hEGF Production in *Saccharomyces cerevisiae*. Fermentation.

[31] Wigneswaran V., Nielsen K.F., Sternberg C., Jensen P.R., Folkesson A., Jelsbak L. (2016). Biofilm as a production platform for heterologous production of rhamnolipids by the non-pathogenic strain *Pseudomonas putida* KT2440. Microb. Cell Fact..

[32] Borges A., Saavedra M.J., Simoes M. (2012). The activity of ferulic and gallic acids in biofilm prevention and control of pathogenic bacteria. Biofouling.

[33] Hall-Stoodley L., Costerton J.W., Stoodley P. (2004). Bacterial biofilms: From the natural environment to infectious diseases. Nat. Rev. Microbiol..

[34] Stewart P.S., Franklin M.J. (2008). Physiological heterogeneity in biofilms. Nat. Rev. Microbiol..

[35] Ibitoye O.B., Ajiboye T.O. (2019). Ferulic acid potentiates the antibacterial activity of quinolone-based antibiotics against *Acinetobacter baumannii*. Microb. Pathog..

[36] Plyuta V., Zaitseva J., Lobakova E., Zagoskina N., Kuznetsov A., Khmel I. (2013). Effect of plant phenolic compounds on biofilm formation by *Pseudomonas aeruginosa*. Apmis.

[37] Tuttle A.R., Trahan N.D., Son M.S. (2021). Growth and Maintenance of *Escherichia coli* Laboratory Strains. Curr. Protoc..

[38] Kang S.Y., Heo K.T., Hong Y.S. (2018). Optimization of Artificial Curcumin Biosynthesis in *E. coli* by Randomized 5′-UTR Sequences To Control the Multienzyme Pathway. ACS Synth. Biol..

[39] Ercan D., Demirci A. (2015). Current and future trends for biofilm reactors for fermentation processes. Crit. Rev. Biotechnol..

[40] Zune Q., Delepierre A., Gofflot S., Bauwens J., Twizere J.-C., Punt P.J., Francis F., Toye D., Bawin T., Delvigne F. (2015). A fungal biofilm reactor based on metal structured packing improves the quality of a Gla::GFP fusion protein produced by *Aspergillus oryzae*. Appl. Microbiol. Biotechnol..

[41] Demirci A., Pongtharangkul T., Pometto A.L., Blaschek H.P., Wang H.H., Agle M.E. (2007). Applications of biofilm reactors for production of value-added products by microbial fermentation. Biofilms in the Food Environment.

[42] Cheng K.C., Demirci A., Catchmark J.M. (2010). Advances in biofilm reactors for production of value-added products. Appl. Microbiol. Biotechnol..

[43] Todhanakasem T. (2017). Developing microbial biofilm as a robust biocatalyst and its challenges. Biocatal. Biotransform..

[44] Yanisch-Perron C., Vieira J., Messing J. (1985). Improved M13 phage cloning vectors and host strains: Nucleotide sequences of the M13mp18 and pUC19 vectors. Gene.

[45] Novagen (1999). pET System Manual.

[46] Studier F.W. (2005). Protein production by auto-induction in high density shaking cultures. Protein Expr. Purif..

[47] Froger A., Hall J.E. (2007). Transformation of plasmid DNA into *E. coli* using the heat shock method. J. Vis. Exp..

[48] Gomes L.C., Silva L.N., Simoes M., Melo L.F., Mergulhao F.J. (2015). *Escherichia coli* adhesion, biofilm development and antibiotic susceptibility on biomedical materials. J. Biomed. Mater. Res. A.

[49] Romeu M.J.L., Dominguez-Perez D., Almeida D., Morais J., Campos A., Vasconcelos V., Mergulhao F.J.M. (2020). Characterization of planktonic and biofilm cells from two filamentous cyanobacteria using a shotgun proteomic approach. Biofouling.

