# Supplementary material for: Engineering Escherichia coli Biofilms for Curcumin Production
Source: Molecules. 2025 May 2;30(9):2031. doi: 10.3390/molecules30092031 (PMC12073880; doi:10.3390/molecules30092031)
Supplement: Supplementary file 1 [file molecules-30-02031-s001.zip › molecules-3458790-supplementary.pdf]

Supplementary Material:

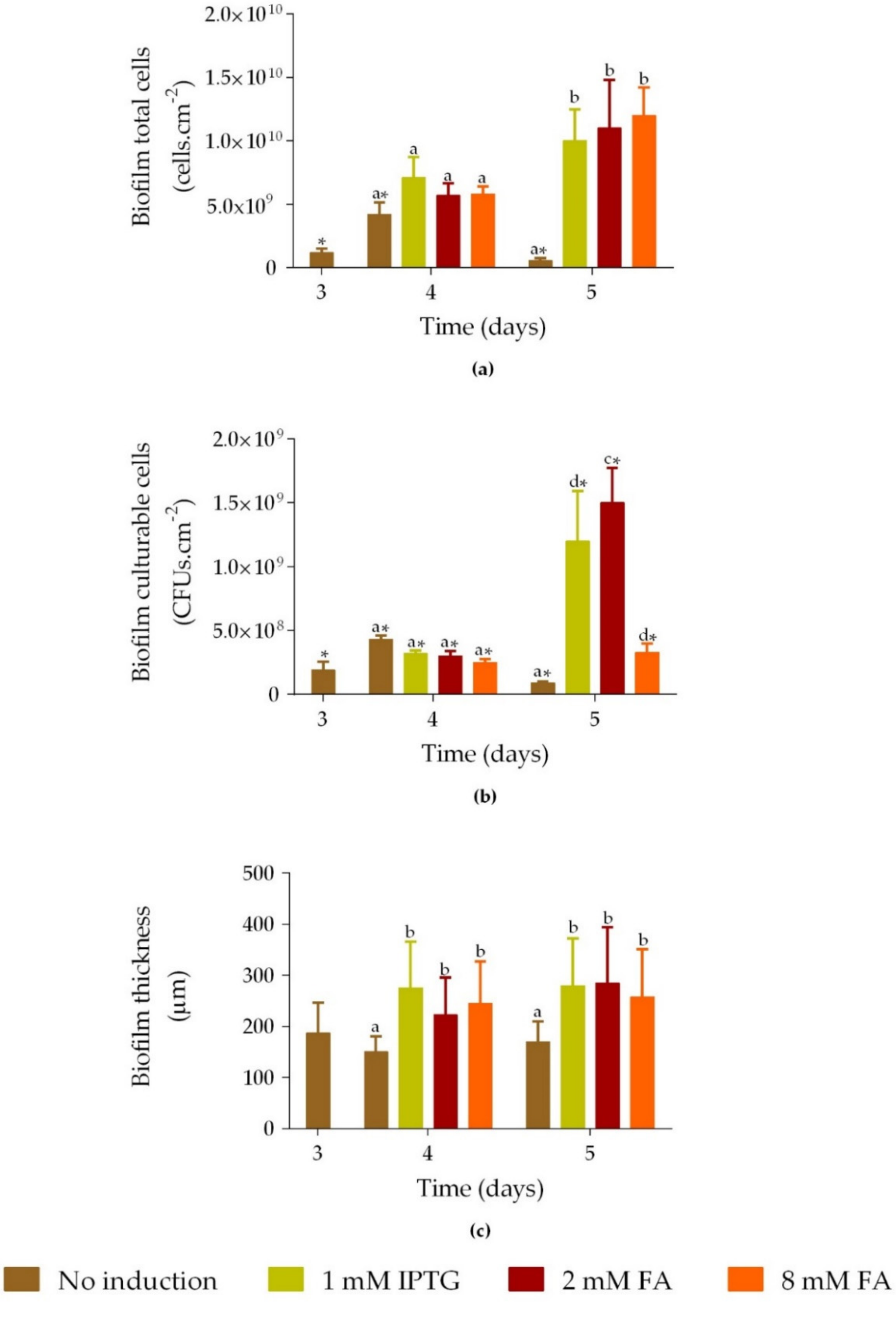

Figure S1. Total and culturable biofilm cells, and biofilm thickness from samples exposed to IPTG only (1 mM) and FA only (2 mM and 8 mM). Samples were incubated at 30 °C without induction (■), and with 1 mM IPTG (■), 2 mM FA (■), and 8 mM FA (■) added on day 3. The samples were analysed on days 3, 4, and 5. The means ± SD for three independent experiments with three technical replicates each are presented. Statistically significant differences within each day (denoted by letters) and between days under the same condition (denoted by *) were considered for p-values < 0.05.

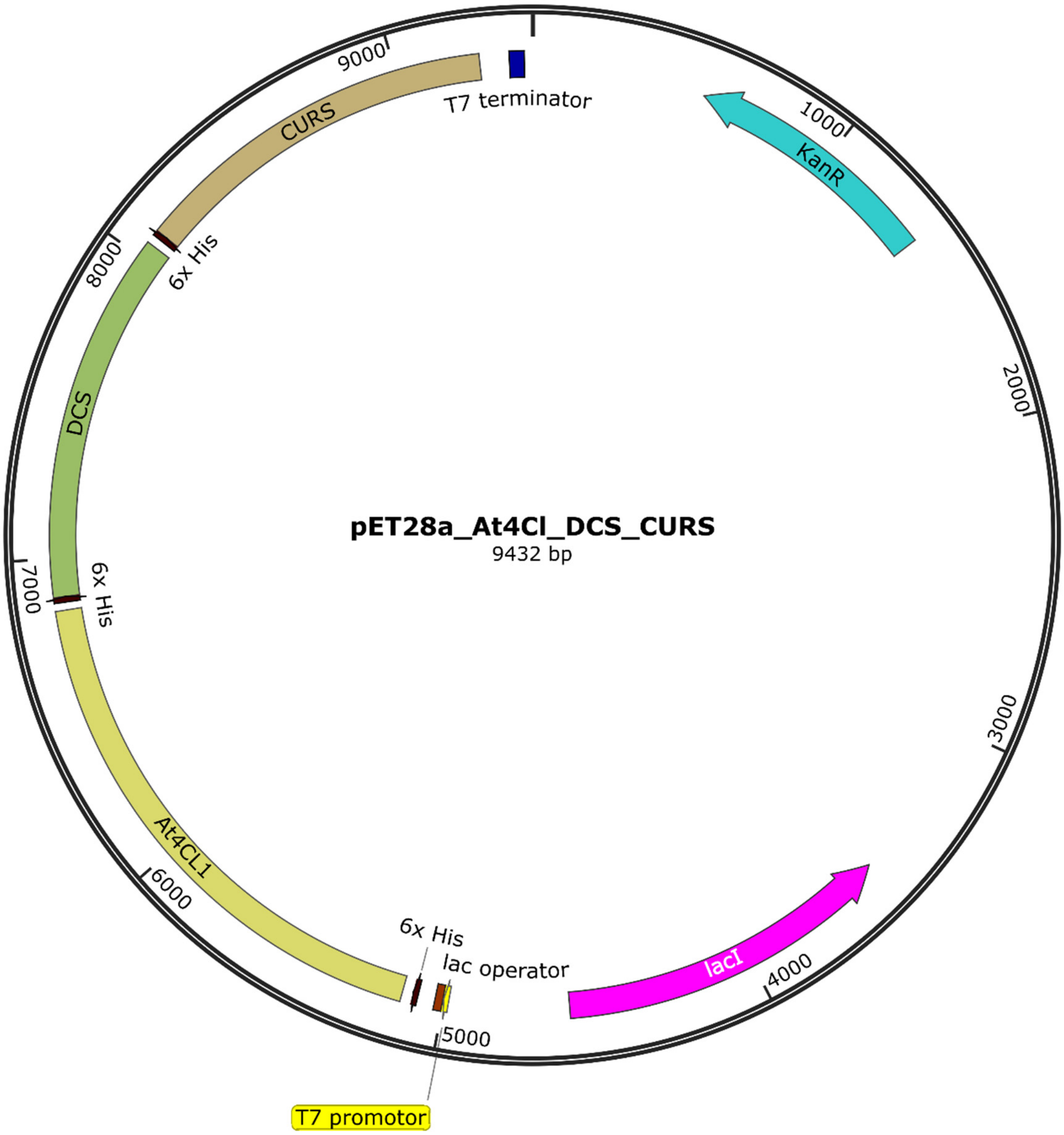

Figure S2. Plasmid At4Cl_DCS_CURS map. This harbors (i) a repressor for the lac promoter (lacI), (ii) a lactose operator (lac operator), (iii) a transcriptional promoter from the T7 phage (T7 promoter), (iv) a T7 transcriptional terminator (T7 terminator), (v) a kanamycin resistance gene (*KanR*), and (vi) the three genes selected for the curcumin biosynthetic pathway: 4-coumarate-CoA ligase (*At4Cl*) from *Arabidopsis thaliana*, and diketide-CoA synthase (*DCS*) and curcumin synthase (*CURS*) from *Curcuma longa*.

Table S1. Residual FA concentration (mM) upon induction with 1 mM IPTG and 8 mM FA or 2 mM FA on day 3 and incubation at 26 °C or 30 °C until day 5. Data are presented as mean ± SD.

**Day**	**8 mM FA**		**2 mM FA**
	26 °C	30 °C	30 °C
**5**	0.013 ± 0.0012	0.576 ± 0.120	1.148 ± 0.310
